# Comparable outcome of liver transplantation with Histidine-Tryptophan-Ketoglutarate vs. University of Wisconsin preservation solution: a retrospective observational double-center trial

**DOI:** 10.1186/1471-230X-14-169

**Published:** 2014-09-28

**Authors:** Alexander Kaltenborn, Jill Gwiasda, Volker Amelung, Christian Krauth, Frank Lehner, Felix Braun, Jürgen Klempnauer, Benedikt Reichert, Harald Schrem

**Affiliations:** Department of General, Visceral and Transplant Surgery, Hannover Medical School, Carl-Neuberg-Str. 1, Hannover, 30625 Germany; Department of Trauma and Orthopedic Surgery, Federal Armed Forces Hospital Westerstede, Westerstede, Germany; Department Health Economics and Health Systems Research of the Institute of Epidemiology, Social Medicine and Health Systems Research, Hannover Medical School, Hannover, Germany; Department of General and Thoracic Surgery, University Hospital Schleswig-Holstein, Kiel, Germany

**Keywords:** Organ procurement, Risk-adjusted analysis, Prolonged cold ischemic time, Biliary complications, Economic evaluation

## Abstract

**Background:**

The question of whether the choice of preservation solution affects outcome after liver transplantation is still not satisfactorily answered. The purpose of this study is to examine the preservation solutions’ impact on outcome after liver transplantation.

**Methods:**

A double-center retrospective study of short- and long-term results of 3134 consecutive liver transplantations with follow-up periods up to 23 years was performed applying multivariate, risk-adjusted analyses with a subset for living-donor transplants, pediatric transplants and cases with prolonged cold ischemic times. An additional focus was put on biliary complications. The primary study endpoints were short- and long-term patient survival and death-censored graft survival. Secondary study endpoints were the occurrence of post-transplant complications, the necessity of operative revisions, the length of hospital stay, and the length of intensive care unit stay.

**Results:**

Although long-term graft survival appears to be increased by Histidine-Tryptophan-Ketoglutarate-use (p = 0.018), this effect could not be confirmed in risk-adjusted analysis (p = 0.641). Multivariate regression analysis revealed that 3-month mortality (p = 0.120), 3-month graft survival (p = 0.103) and long-term patient survival (p = 0.235) were not influenced by the choice of preservation solution. There was no difference in the occurrence of common complications or necessity of operative revisions after liver transplantation. This was confirmed in subgroup analyses for living donor and pediatric transplantation and cases with prolonged cold ischemic time. Analysis of the preservation solutions’ impact on length of hospital (p = 0.113) and intensive care unit stay (p = 0.481) revealed no significant difference.

**Conclusions:**

University of Wisconsin and Histidine-Tryptophan-Ketoglutarate solutions are clinically equivalent. Histidine-Tryptophan-Ketoglutarate solution could have an economically superior profile. The notion that the choice of preservation solution can have an impact on the onset of biliary complications after liver transplantation remains a matter of controversy.

**Electronic supplementary material:**

The online version of this article (doi:10.1186/1471-230X-14-169) contains supplementary material, which is available to authorized users.

## Background

University of Wisconsin (UW) and Histidine-Tryptophan-Ketoglutarate (HTK) solutions are both used for organ preservation in liver transplantation (LTX). Both have been applied for decades largely depending on local preferences with several known differences. UW is characterized by low osmolarity, the containment of colloid and several radical scavengers, such as adenosine and glutathione [1]. It revolutionized organ preservation in 1987 by facilitating “semi-elective” LTX [2]. HTK was developed by Bretschneider and colleagues in 1975, originally for cardioplegia [1]. In 2002, the US Food and Drug Administration approved HTK for preservation of donor livers. It is characterized by a lower osmolarity than UW, a strong histidine buffer system which increases the osmotic effect of mannitol and lower electrolyte concentrations [1, 3].

The effects of the choice of preservation solution (PS) on outcome after organ transplantation are still a matter of controversy. A systematic review of reports published until 2007 with a total of 1200 liver transplant recipients states that both are equally effective [4]. More recent data from the UNOS database points to preferable characteristics of UW, especially in means of graft survival [5]. Furthermore, the impact of PS on the onset of biliary complications still remains unclear, since there are reports suggesting equivalent [6, 7] as well as differing risk profiles [8–10]. However, most of the available literature is limited by its single-center study character, small sample sizes and only short- to medium-term follow-up schemes.

We performed a double-center, data-base driven statistical analysis of the short- and long-term results of more than 3100 liver transplant recipients. Narrowly scoped subset-analyses were performed additionally for living-donor transplants, pediatric transplants, as well as cases with prolonged cold ischemic time (CIT). Additionally, a focused investigation of biliary complications was part of this analysis.

## Methods

This is a double-center, retrospective, observational study from two German liver transplant centers within the Eurotransplant community. The study was designed according to the Strengthening the Reporting of Observational Studies in Epidemiology (STROBE)-Guidelines (see STROBE-checklist as Additional file 1). Hannover Medical School provided data of 2554 consecutive liver transplants (1377 males [53.9%]; 1177 females [46.1%]) with a median recipient age of 43 years at transplant (range 0-74 years) performed between the 01.06.1987 and the 31.12.2012. Mean follow-up in this cohort is 5.7 years (median 3.9 years; range 0-22 years). The University Hospital Kiel provided data of 580 consecutive transplants (336 males [57.9%]; 244 females [42.1%]) with a median recipient age of 52 years at transplant (range 0-75 years) performed between the 01.11.1988 and the 09.02.2013. Mean follow-up in this cohort is 3.1 years (median 3.2; range 0-23.3 years). Only patients with either HTK or UW usage were included. Cases with missing data over more than 10% of follow-up time were excluded from analyses. The primary study endpoints were short- and long-term patient survival as well as death-censored graft survival. Short-term patient and graft survival was defined as 90 days post-transplant. The secondary study endpoints were the occurrence of post-transplant complications (hepatic artery thrombosis (HAT), portal vein thrombosis (PVT), caval vein thrombosis, post-operative hemorrhage, biliary complications), the necessity of operative revisions, the length of hospital stay, and the length of intensive care unit (ICU) stay.

### Ethical considerations

As an observational retrospective study, according to the Professional Code of the German Medical Association (article B.III. § 15.1), neither informed consent nor approval of the ethics committee was needed for this study. This study is in compliance with the Helsinki Declaration.

### Statistical methods

This is an analysis of prospectively stored and retrospectively compiled data. Age at transplant, recipient sex, allocation era (I: early phase center allocation 01/01/1987-31/12/1992; II: late phase center allocation 01/01/1993-30/06/2000; III: Child-Pugh-based allocation system 01/07/2000-01/01/2007; IV: Model of End-Stage Liver Disease-based allocation 01/01/2007-09/02/2013), living-donor transplant, experience of the operating surgeon (defined as previously performed LTX as leading surgeon), CIT, pediatric patient (defined as younger than 18 years), operative duration, combined transplant, number of post-operative revisions, complications (as listed above), as well as the cohort’s five most common indications leading to transplant (see Table 1) were analyzed as possible risk factors with influence on the primary and secondary study endpoints. Their relevance was identified with univariate binary regression and Cox regression analysis respectively. To avoid over-fitting, all variables with an alpha-level <0.1 were considered for risk-adjusted multivariate regression analyses, which were performed to examine variations in risk according to usage of either UW or HTK solution. Kaplan-Meier analysis with Log Rank Tests, Pearson’s Chi^2^-test or ANOVA-analysis were applied where appropriate. For all statistical tests a p-value <0.05 was defined as significant. The IBM SPSS statistics-software version 21.0 was used to perform statistical analysis.

**Table 1:** **The five most common indications for liver transplantation in the Hannover and Kiel cohort**

	Hannover cohort		Kiel cohort	
	Indication	Number of patients (% of cohort)	Indication	Number of patients (% of cohort)
**1**	Viral hepatitis	334 (13.0%)	Alcoholic cirrhosis	119 (20.5%)
**2**	Hepatocellular carcinoma	281 (11.0%)	Viral hepatitis	86 (14.8%)
**3**	Acute liver failure	246 (9.6%)	Hepatocellular carcinoma	43 (7.4%)
**4**	Primary sclerosing cholangitis	196 (7.7%)	Biliary atresia	41 (7.1%)
**5**	Biliary atresia	192 (7.5%)	Acute liver failure	36 (6.2%)

### Additional subset analysis

An additional detailed subset investigation was performed for the Hannover cohort’s living donor related transplants (n = 125; 4.9%), pediatric patients (n = 575; 22.5%), and patients with prolonged CIT (n = 346; 13.5%). Prolonged CIT was defined as the mean CIT (622 min) plus the standard deviation (239 min) which results in a CIT > 861 min (the CIT was normally distributed, Kolmogorov-Smirnov-Test: p < 0.001).

### Amount of preservation solution

The Kiel data-base stored data on the perfused amounts of UW and HTK solution for 507 patients (87.4%). This data was investigated as a continuous variable in risk-adjusted regression modeling to assess its influence on the primary and secondary study endpoints. We aimed to reveal a potential dose-dependent effect of the preservation solution on outcome with this analysis. Furthermore, addition of the Kiel cohort served to confirm the results from the Hannover analysis and aimed to reduce possible center bias.

### Clinical handling of UW and HTK

Before application, several additives, such as prostaglandin E1 and/or dexamethason have to be added to UW solution. Once these components have been mixed, the solution must be used within 24h. In contrast, HTK solution can be used right away and no further additives are needed. From a clinical point of view, HTK can be quicker perfused without any need for pressurized perfusion. This is due to HTK’s comparatively low viscosity, which equals the one of water (2.0 cP). The viscosity of UW is higher (6.2 cP) because of the presence of colloids [1, 11].

## Results

**Figure 1:**
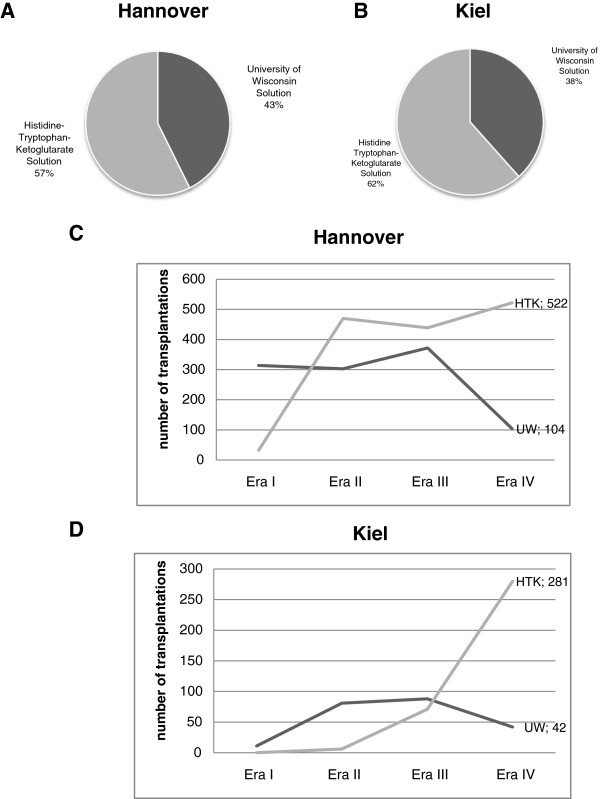
**Distribution of preservation solution in the Hannover (A) and Kiel (B) cohort as well as usage of solutions over the follow-up time in the Hannover (C) and Kiel cohort (D).**

Descriptive statistics of the two cohorts and the distribution of UW vs. HTK solution are summarized in Tables 2, 3 and 4. In Hannover, UW was used in 1091 transplants (42.7%) and HTK in 1463 transplants (57.3%). In Kiel, livers were perfused 223 times with UW (38.4%) and 375 times with HTK (61.6%). The distribution and development of solution usage above time is depicted in Figure 1.

**Table 2:** **Distribution of primary and secondary study endpoints as well as preservation solutions to the different eras, 2554 patients from the Hannover liver transplant program**

	Era				
	I	II	III	IV	p-value
n (%)	346 (13.5%)	773 (30.3%)	811 (31.8%)	624 (24.4%)	n.a.
HTK	32 (9.2%)	470 (60.8%)	439 (54.1%)	522 (83.7%)	<0.001^1^
UW	314 (90.8%)	303 (39.2%)	372 (45.9%)	102 (16.3%)	<0.001^1^
3 months mortality	82 (23.7%)	142 (18.4%)	112 (13.8%)	98 (15.7%)	<0.001^1^
graft loss	224 (64.7%)	374 (48.4%)	281 (34.6%)	171 (27.4%)	<0.001^1^
3 month graft survival	238 (68.8%)	582 (75.3%)	645 (79.5%)	482 (77.4%)	0.001^1^
HAT	14 (4.0%)	19 (2.5%)	30 (3.8%)	37 (6.0%)	0.010^1^
Portal vein thrombosis	6 (1.9%)	7 (0.9%)	24 (3.1%)	27 (4.4%)	0.001^1^
Caval vein thrombosis	1 (0.3%)	4 (0.5%)	10 (1.3%)	31 (5.0%)	<0.001^1^
Post-operative hemorrhage	73 (22.7%)	197 (25.9%)	162 (20.8%)	169 (27.3%)	0.020^1^
Biliary complications	34 (16.9%)	127 (16.7%)	169 (21.7%)	138 (22.3%)	0.015^1^
Mean LOS in days (SD)	44 (34)	40 (30)	41 (35)	51 (43)	<0.001^2^

HTK = Histidine-Tryptophane-Ketoglutarate solution; UW = University of Wisconsin solution; SD = Standard Deviation; HAT = hepatic artery thrombosis; LOS = length of hospital stay.

^1^Pearson’s Chi^2^ Test.

^2^ANOVA.

**Table 3:** **Distribution of primary and secondary study endpoints as well as preservation solutions to the different eras, 580 patients from the Kiel liver transplant program**

	Era				
	I	II	III	IV	p-value
n (%)	9 (1.6%)	96 (16.6%)	152 (26.2%)	323 (55.7%)	n.a.
HTK	0 (0%)	8 (8.3%)	68 (44.7%)	281 (87.0%)	<0.001^1^
UW	9 (100.0%)	88 (91.7%)	84 (55.3%)	42 (13%)	<0.001^1^
3 months mortality	3 (33.3%)	22 (22.9%)	35 (23.0%)	32 (9.9%)	<0.001^1^
graft loss	8 (88.9%)	59 (61.5%)	75 (49.7%)	85 (26.5%)	<0.001^1^
3 month graft survival	6 (66.7%)	68 (70.8%)	106 (69.7%)	262 (81.1%)	0.021^1^
Biliary complications	1 (11.1%)	33 (34.4%)	47 (30.9%)	18 (19.4%)	0.099^1^

HTK = Histidine-Tryptophane-Ketoglutarate solution; UW = University of Wisconsin solution; SD = Standard Deviation.

^1^Pearson’s Chi^2^ Test.

**Table 4:** **Shown are clinical characteristics of the two investigated cohorts from Hannover Medical School and University Hospital Kiel**

Variable	Hannover cohort	Kiel cohort
n	2554	580
sex	1377 males (53.9%); 1177 females (46.1%)	336 males (57.9%); 244 females (42.1%)
Median age (range)	43 (0-74) years	52 (0-75) years
UW (n; %)	1091 (42.7%)	223 (38.4%)
HTK (n; %)	1463 (57.3%)	375 (61.6%)
CIT (mean; range) in min.	621.6 (80-1970)	601.5 (210-1320)
Living donor (n; %)	125 (4.9%)	80 (13.8%)
Pediatric recipient (n; %)	575 (22.5%)	87 (15.0%)

HTK = Histidine-Tryptophane-Ketoglutarate solution; UW = University of Wisconsin solution; CIT = cold ischemic time.

### The Hannover cohort

#### Patient survival

**Figure 2:**
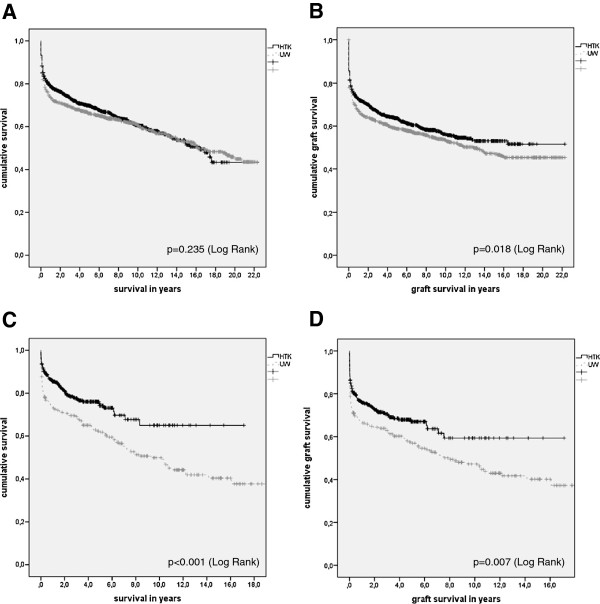
**Results of univariate Kaplan Meier analysis of patient (A) and graft (B) survival distributed by the preservation solution in the Hannover cohort as well as patient (C) and graft (D) survival in the Kiel cohort.**

In Kaplan-Meier analysis the type of PS has no statistically significant impact on long-term patient survival (p = 0.235) (Figure 2A). Short-term patient survival was not significantly affected by the type of preservation solution (p = 0.120).

#### Graft survival

Univariate Kaplan-Meier analysis demonstrated that HTK is beneficial for long-term graft survival (p = 0.018; Log-Rank) (Figure 2B). This result could not be confirmed in multivariate Cox regression (p = 0.641), which was adjusted for necessity of operative revisions (p < 0.001; HR:1.943; 95%-CI:1.711-2.206), occurrence of HAT (p < 0.001; HR:4.306; 95%-CI:3.447-5.379), PVT (p < 0.001; HR:1.973; 95%-CI:1.414-2.784), post-operative hemorrhage (p < 0.001; HR:1.540; 95%-CI:1.346-1.763), biliary complications (p = 0.003; HR:1.260; 95%-CI:1.086-1.461), age at transplant (p < 0.001; HR:1.007; 95%-CI:1.004-1.011), living-donor LTX (p < 0.001; HR:0.539; 95%-CI:0.370-0.785), CIT (p < 0.001; HR:1.001; 95%-CI:1.000-1.001), pediatric LTX (p = 0.001; HR:0.775; 95%-CI:0.662-0.908), era in which the transplant was performed (p < 0.001; HR:0.811; 95%-CI:0.760-0.866), the indication hepatocellular carcinoma (HCC) (yes/no) (p = 0.022; HR:1.247; 95%-CI:1.038-1.498), the indication primary sclerosing cholangitis (PSC) (yes/no) (p = 0.001; HR:0.651; 95%-CI:0.501-0.847) and the indication biliary atresia (yes/no) (p = 0.007; HR:0.702; 95%-CI:0.535-0.921). Three-month graft survival was not affected by the type of PS (p = 0.103).

#### Complications after LTX

The choice of PS did not influence the occurrence of HAT (p = 0.850), nor of PVT (p = 0.761), caval vein thrombosis (p = 0.053), post-operative hemorrhage (p = 0.273), biliary complications (p = 0.178) and necessity of operative revisions (p = 0.120) after LTX.

Biliary complications (n = 488; 19.1%) have been further analyzed on a more detailed level, because of previously reported associations of these with the PS [11–13]. They were categorized into biliary leaks (n = 218; 44.7%), stenosis (n = 77; 15.8%), cholangitis (n = 80; 16.4%) and not further specified biliary complications (n = 129; 26.4%). In univariate regression analysis, beneficial effects on biliary leakage (p = 0.035; OR:0.735; 95%-CI: 0.531-0.982) and biliary stenosis (p = 0.011; OR:0.532; 95%-CI: 0.323-0.879) were associated with HTK-preservation. This result for biliary leakage could not be verified in a multivariate binary regression model (p = 0.572), which was adjusted for the occurrence of HAT (p = 0.001; OR:2.566; 95%-CI:1.525-4.317), caval thrombosis (p = 0.043; OR:2.227; 95%-CI:1.025-4.835), post-operative hemorrhage (p = 0.001; OR:1.690; 95%-CI:1.256-2.274), duration of LTX-procedure (p = 0.011; OR:0.998; 95%-CI:0.997-1.000), living-donor LTX (p < 0.001; OR:2.826; 95%-CI:1.779-4.489), CIT (p < 0.001; OR:0.999; 95%-CI:0.998-0.999), the use of hepaticojejunostomy (p < 0.001; OR:2.146; 95%-CI:1.618-2.848), PVT (p = 0.012; OR:0.439; 95%-CI:0.213-0.905), pediatric LTX (p = 0.005; OR:1.571; 95%-CI:1.156-2.136), era in which the transplant was performed (p < 0.001; OR:1.584; 95%-CI:1.360-1.894), the indications viral hepatitis (yes/no) (p = 0.001; OR:0.410; 95%-CI:0.231-0.727), acute liver failure (yes/no) (p = 0.021; OR:0.524; 95%-CI:0.288-0.953) and biliary atresia (yes/no) (p < 0.001; OR:2.251; 95%-CI:1.489-3.405). Moreover, the beneficial influence of HTK on the rate of biliary stenosis could not be confirmed in a multivariate regression model (p = 0.122), which was risk-adjusted for the occurrence of HAT (p < 0.001; OR:5.398; 95%-CI:2.865-10.171) and the era in which the transplant was performed (p = 0.001; OR:1.486; 95%-CI:1.162-1.899). Analysis of cases with post-transplant cholangitis (p = 0.353) as well as not specified biliary complications (p = 0.254) remained insignificant.

#### Length of hospital and ICU stay

Neither the length of hospital stay (p = 0.113) nor the length of ICU stay (p = 0.481) after LTX were affected by the choice of PS.

#### Pediatric and living donor transplant

22.5% of the Hannover cohort were pediatric patients (n = 575). HTK was used more often in this subgroup (n = 302; 52.5%) than UW (n = 273; 47.5%) (p < 0.001; Chi^2^). None of the solutions influenced long-term patient (p = 0.104) and graft survival (p = 0.059) or three-month mortality (p = 0.056) and three-month graft survival (p = 0.126). In the pediatric subgroup, the onset of biliary complications was not influenced by the choice of PS (p = 0.059).

125 patients (4.9%) received a living-donor related transplant during follow-up. In 114 cases (91.2%) a HTK-preserved organ was transplanted; in 11 cases (8.8%) UW-solution was used (p < 0.001; Chi^2^). None of the solutions influenced long-term patient (p = 0.125) and graft survival (p = 0.085) or three-month mortality (p = 0.258) and three-month graft survival (p = 0.181). 33 patients (26.4%) developed biliary complications after living-donor related LTX. All of these transplanted grafts had been conserved with HTK, thus there is a statistically significant association between HTK-use and biliary complications in this sub-cohort (p = 0.036).

#### Prolonged cold ischemic time

Definition of prolonged CIT is outlined in the Methods section. 346 (13.5%) patients received an organ that was exposed to a prolonged CIT. 116 of these (33.5%) donor organs have been preserved with HTK and 230 (66.5%) using UW (p < 0.001; Chi^2^). In this sub-cohort, the choice of PS had no statistically significant influence on long-term patient (p = 0.989) and graft (p = 0.796) survival as well as three-month mortality (p = 0.550) and three-month graft survival (p = 0.724).

Furthermore, there was no statistically significant difference between the rate of biliary complications in the two PS cohorts (p = 0.097).

### The Kiel cohort

In Kiel, 223 patients (38.4%) received a UW-preserved organ and 375 patients (61.6%) a HTK-preserved donor liver (see Figure 1B). Part of this cohort were 80 living donor related LTX (13.8%) and 87 pediatric recipients (15%). The mean CIT was 602 minutes (SD: 152 minutes). A subset analysis equivalent to the Hannover cohort was performed for the Kiel cohort without significantly differing results (data not shown). It was thus decided to report the Hannover cohort’s results, since this cohort appears to be the larger group of patients.In univariate Kaplan Meier analysis, patient (Figure 2C; p < 0.001; Log Rank) as well as graft (Figure 2D; p = 0.007; Log Rank) survival were associated with the choice of PS. However, long-term patient (p = 0.798) and graft survival (p = 0.454) were not influenced by the PS in multivariate analysis (adjusted for living donor transplantation, number of operative revisions, recipient age at transplant and era; all p < 0.001). Investigation of short-term outcome also revealed no significant results in risk-adjusted regression analysis (short-term mortality: p = 0.576; short-term graft survival: p = 0.396). The PS had no influence on the occurrence of biliary complications (p = 0.728) or the necessity of operative revisions (p = 0.256).

An average of 4,869 ml UW solution was used for donor organ preservation (median 5,000 ml; range 1,500-10,000 ml). HTK preservation needed higher amounts of averaged 8,303 ml (median 8,000; range 1,000-17,000). Neither the amount of UW nor of HTK was associated with three-month mortality (UW: p = 0.518; HTK: p = 0.417) or graft survival (UW: p = 0.789; HTK: p = 0.053), biliary complications (UW: p = 0.518; HTK: p = 0.955) as well as long-term patient (UW: p = 0.395; HTK: p = 0.021, HR:1.000) or graft survival (UW: p = 0.454; HTK: p = 0.026, HR:1.000).

#### Economical evaluation

The costs for HTK per liter range between 50 and 56 Euro based on the amount of ordered units in Germany. There is currently no governmental approval for UW, thus no UW solution available on the German market. We therefore retrieved the costs of UW per liter from the literature, where costs of 181 Dollar per liter (≈136 Euro) are stated for UW on the US market [1]. Based on this information and taking the mean amount of PS into account, preservation of an average liver graft with HTK costs 415 Euro, whereas an average UW-perfused graft costs 662 Euro. Furthermore, the costs for the said additives for preparation of UW solution have to be taken into account.

## Discussion

Both HTK and UW are widely used for graft preservation in solid organ transplantation [4, 14, 15]. There are several studies available on the influence of the PS on outcome defined as survival, onset of complications (especially in the biliary tract) and rejection rates [4, 5, 14, 15]. The results of these studies range from equality of the solutions [4] to favoring one or the other [5, 10], thus the evidence-based choice of preservation solution remained a matter of controversy. Our local clinical situation is actually sharpened by the concerning fact that UW has currently no valid license for organ preservation and is thus not available in Germany. This has become problematic, since there are organs donated in other Eurotransplant partner nations, e.g. Belgium, where UW is still approved and used, leaving German transplant physicians without the possibility to re-perfuse an organ flown in from these countries. We recently experienced such an unfortunate and evitable event. A clinical, evidence-based standard to support the choice of PS use is thus indispensable.

We therefore performed an analysis with the data of two liver transplant centers (Hannover and Kiel, Germany), which are both within the Eurotransplant community and applied widely established statistical methods rigorously to compare the preservation solutions’ influence on short- and long-term patient and graft survival, complications after LTX as well as the length of hospital and ICU stay. Competing risks were identified as possible confounders and systematically taken into account in multivariate analysis. An advantage of this study is the large number of included cases (total n = 3134), which represents, at least to our knowledge, the largest non-registry-based investigation of this kind available at the moment.

We believe that including both analyses of the Hannover and Kiel cohorts significantly improves the grade of information of the manuscript. On the one hand, the major relevant data, e.g. on patient and graft survival or the PS applied is given in both datasets and was investigated to reveal the PS’s impact on outcome and reduce the possible center bias. On the other hand, interesting data, such as the amount of perfused PS or the secondary study endpoints like post-transplant complications was only available from one of the two co-operating transplant centers. Nevertheless, this is relevant information; therefore, the combination of the results in this manuscript seems warranted.Short- and long-term patient survival was not influenced by the choice of PS. Although long-term graft survival seemed to be increased by HTK-use (see Figure 2A), this effect could not be confirmed in risk-adjusted analysis. Furthermore, 3-month graft survival did not differ in the two PS cohorts. Since no association between the PS and common complications could be shown, we assume that HTK and UW are clinically equivalent choices and no PS can be preferred in terms of influence on the short- or long-term outcome after LTX. This can be confirmed for living donor as well as pediatric LTX. The data further revealed that it does not matter how much solution is actually used for preservation, as long the amount seems clinically sufficient (the investigated amounts of PS range from 1,500-10,000 ml UW and 1,000-17,000 ml HTK).

To answer the question whether one of the solutions is preferable in situations with extensively prolonged CIT, this subgroup was defined, identified and subsequently analyzed. Also in this subset analysis the choice of PS made no statistically significant difference regarding short- and long-term patient and graft survival as well as the onset of post-transplant complications.

The notion that the choice of PS can have an impact on the onset of biliary complications after LTX is still a matter of controversy [6–10]. This aspect was therefore analyzed in depth resulting in no statistically significant difference between UW and HTK in means of onset of either biliary leakage (p = 0.572), stenosis (p = 0.122), post-transplant cholangitis (p = 0.353) and not further specified biliary complications (p = 0.254). The result that there might be more biliary complications after living-donor LTX is probably biased by the overall larger number of HTK-perfused grafts in this subgroup (HTK-use in 91.2%). Another possible explanation for this observation may be caused by differing practices in perfusion of the segment IV artery and the segment IV bile duct with PS. Furthermore, it is known that partial liver grafts are associated with increased bile duct leaks from the liver resection surface.

Since our data suggests that there is no clinical difference between UW and HTK in LTX, economic considerations can play an important role for the choice of PS. As one important economical factor we analyzed the PS’s impact on length of hospital and ICU stay, which was both not affected by the PS. HTK is clinically easier to handle, because it is sold in ready-to-use units while UW must be mixed with additives prior to use, although this might not be done anymore in some centers without deteriorating outcome [1]. Furthermore, the quicker perfusion of the graft with HTK compared to UW can be seen as cost-benefit advantage (“time is money”). However, this effect is reduced by the fact that in average twice the amount of HTK is flushed. The routine use of expensive medications, including the PS, has been identified as one driving factor of cost-increase in transplantation [16]. The costs per liter vary drastically between the two investigated PS leading to the conclusion that HTK is the solution with the economically superior profile. A study aiming to find a cost prediction model for LTX from 2008 [17] nevertheless reported that the median total cost of LTX significantly differs regarding two cohorts with either UW or HTK usage in favor of UW, although this result was not validated by a multivariate analysis. Also the number of included patients was smaller (n = 139) than our study-population. Further investigations of these important economic aspects are needed to fully understand these interesting findings.

In conclusion, it is hard to really determine the economical impact of the PS, especially in the setting of this study. Due to center volume and individual negotiations there are different prices even among centers in the same region or country.

This study is limited due to the fact that non heart-beating donation is legally prohibited in Germany and thus no such cases could be included. This aspect might be partly responsible for the discrepancy between our results and the UNOS-registry study by Stewart et al. from 2009 [5], in which grafts donated after cardiac death were included.

It is somehow surprising that the rates of HAT and PVT are currently increasing as compared to the previous eras (see Table 2), which might be due to the improved diagnostic capacities and thus higher detection rates rather than worsening results. Another important aspect in this context is the notion that with the introduction of MELD-based liver allocation and the “sickest-first-principle” in Germany, patients accepted for a transplant tend to be sicker nowadays than in earlier eras [18, 19]. This might be also reflected in the increasing lengths of hospital and ICU stay (see Table 2). Deteriorating outcome and/or stagnating progress in LTX, especially for some indications such as PSC, is currently under hot debate in Germany and the shown data (Tables 2 and 3) underlines this observation [20], which is in sharp contrast to improved outcome recently reported from a large US series [21].

Recently published data on a biochemically improved HTK solution may provide a promising option for the future [22]. Liu and colleagues reported that HTK-N solution, which is modified with N-actylhistidine, amino-acids and iron chelators, protects liver grafts with microvesicular steatosis better than common HTK in a rat model [22]. This result is especially interesting given the expanding use of extended donor criteria in LTX. Celsior solution might also be able to show some advantages, which was underlined in a recent prospective multicenter trial from France [23]. Further studies are needed, preferably with higher levels of evidence such as prospective multicenter trials or randomized registry trials to investigate the preliminary results of our study. In times of chronically diminishing donation rates in Germany [19], it is the transplant society’s responsibility to ensure the best possible preservation of the scarce donor organs. Thus, further investigations regarding this topic are warranted.

## Conclusions

UW and HTK are clinically equivalent. HTK could have an economically superior profile. However, it is hard to really determine the economical impact of the PS, especially in the setting of this study. The notion that the choice of preservation solution can have an impact on the onset of biliary complications after liver transplantation remains a matter of controversy. All authors read and approved the final manuscript.

## Authors’ information

Benedikt Reichert and Harald Schrem, both authors share last authorship.

## Electronic supplementary material

Additional file 1: **STROBE Statement—Checklist of items that should be included in reports of cohort studies.** (DOC 98 KB)

## Abbreviations

CIT: Cold ischemic time
HAT: Hepatic artery thrombosis
HCC: Hepatocellular carcinoma
HR: Hazard ratio
HTK: Histidine-Tryptophane-Ketoglutarate solution
ICU: Intensive care unit
LOS: Length of hospital stay
LTX: Liver transplantation
OR: Odds ratio
PS: Preservation solution
PSC: Primary sclerosing cholangitis
PVT: Portal vein thrombosis
SD: Standard deviation
UW: University of Wisconsin solution
95%-CI: 95%-confidence interval.

## References

[1] Bellamy CA, Nicely B, Mattice BJ, Teaster R (2008). Comparative analysis of clinical efficacy and cost between University of Wisconsin solution and histidine-tryptophan-ketoglutarate. Prog Transplant.

[2] Guarrera JV, Karim NA (2008). Liver preservation: is there anything new yet?. Curr Opin Organ Transplant.

[3] Fridell JA, Agarwal A, Milgrom ML, Goggins WC, Murdock P, Pescovitz MD (2004). Comparison of histidine-tryptophan-ketoglutarate solution and University of Wisconsin solution for organ preservation in clinical pancreas transplantation. Transplantation.

[4] Feng L, Zhao N, Yao X, Sun X, Du L, Diao X, Li S, Li Y (2007). Histidine-tryptophan-ketoglutarate solution vs. University of Wisconsin solution for liver transplantation: a systematic review. Liver Transpl.

[5] Stewart ZA, Cameron AM, Singer AL, Montgomery RA, Segev DL (2009). Histidine-Tryptophan-Ketoglutarate (HTK) is associated with reduced graft survival in deceased donor livers, especially those donated after cardiac death. Am J Transplant.

[6] Rayya F, Harms J, Martin AP, Bartels M, Hauss J, Fangmann J (2008). Comparison of histidine-tryptophan-ketoglutarate solution and University of Wisconsin solution in adult liver transplantation. Transplant Proc.

[7] Moench C, Otto G (2006). Ischemic type biliary lesions in histidine-tryptophan-ketoglutarate (HTK) preserved liver grafts. Int J Artif Organs.

[8] Meine MH, Zanotelli ML, Neumann J, Kiss G, De Jesus Grezzana T, Leipnitz L, Schlindwein ES, Fleck A, Gleisner AL, de Mello Brandão A, Marroni CA, Cantisani GP (2006). Randomized clinical assay for hepatic grafts preservation with University of Wisconsin or histidine-tryptophan-ketoglutarate solutions in liver transplantation. Transplant Proc.

[9] Welling TH, Heidt DG, Englesbe MJ, Magee JC, Sung RS, Campbell DA, Punch JD, Pelletier SJ (2008). Biliary complications following liver transplantation in the model for end-stage liver disease era: effect of donor, recipient, and technical factors. Liver Transpl.

[10] Mangus RS, Tector AJ, Agarwal A, Vianna R, Murdock P, Fridell JA (2006). Comparison of histidine-tryptophan-ketoglutarate solution (HTK) and University of Wisconsin solution (UW) in adult liver transplantation. Liver Transpl.

[11] Chan SC, Liu CL, Lo CM, Fan ST (2004). Applicability of histidine-tryptophan-ketoglutarate solution in right lobe adult-to-adult live donor liver transplantation. Liver Transp.

[12] Canelo R, Hakim NS, Ringe B (2003). Experience with hystidine tryptophan ketoglutarate versus University Wisconsin preservation solutions in transplantation. Int Surg.

[13] Erhard J, Lange R, Scherer R, Kox WJ, Bretschneider HJ, Gebhard MM, Eigler FW (1994). Comparison of histidine-tryptophan-ketoglutarate (HTK) solution versus University of Wisconsin (UW) solution for organ preservation in human liver transplantation. A prospective randomized study. Transplant Int.

[14] Jamieson RW, Friend PJ (2008). Organ reperfusion and preservation. Front Biosci.

[15] Wilson CH, Brook NR, Talbot D (2006). Preservation solutions for solid organ transplantation. Mini Rev Med Chem.

[16] Portela MP, Neri ED, Fonteles MM, Garcia JH, Fernandes ME (2010). The cost of liver transplantation at a university hospital of Brazil. Rev Assoc Med Bras.

[17] Earl TM, Cooil B, Rubin JE, Chari RS (2008). Cost prediction in liver transplantation using pretransplant donor and recipient characteristics. Transplantation.

[18] Weismüller TJ, Fikatas P, Schmidt J, Barreiros AP, Otto G, Beckebaum S, Paul A, Scherer MN, Schmidt HH, Schlitt HJ, Neuhaus P, Klempnauer J, Pratschke J, Manns MP, Strassburg CP (2011). Multicentric evaluation of model for end-stage liver disease-based allocation and survival after liver transplantation in Germany–limitations of the ‘sickest first’-concept. Transpl Int.

[19] Schrem H, Kaltenborn A (2013). Germany: Avoid more organ transplant scandals. Nature.

[20] Klose J, Klose MA, Metz C, Lehner F, Manns MP, Klempnauer J, Hoppe N, Schrem H, Kaltenborn A (2014). Outcome stagnation of liver transplantation for primary sclerosing cholangitis in the Model for End-Stage Liver Disease era. Langenbecks Arch Surg.

[21] Agopian VG, Petrowsky H, Kaldas FM, Zarrinpar A, Farmer DG, Yersiz H, Holt C, Harlander-Locke M, Hong JC, Rana AR, Venick R, McDiarmid SV, Goldstein LI, Durazo F, Saab S, Han S, Xia V, Hiatt JR, Busuttil RW (2013). The evolution of liver transplantation during 3 decades: analysis of 5347 consecutive liver transplants at a single center. Ann Surg.

[22] Liu Q, Bruns H, Schultze D, Xue Y, Zorn M, Flechtenmacher C, Straub BK, Rauen U, Schemmer P (2012). HTK-N, a modified HTK solution, decreases preservation injury in a model of microsteatotic rat liver transplantation. Langenbecks Arch Surg.

[23] Boudjema K, Grandadam S, Compagnon P, Salamé E, Wolf P, Ducerf C, Le Treut P, Soubrane O, Cherqui D, Mouchel C, Renault A, Bellissant E (2012). Efficacy and safety of Celsior preservation fluid in liver transplantation: one-year follow up of a prospective, multicenter, non-randomized study. Clin Transplant.

[CR24] The pre-publication history for this paper can be accessed here:http://www.biomedcentral.com/1471-230X/14/169/prepub

